# Molecular characterization of cytidine monophospho-N-acetylneuraminic acid hydroxylase (CMAH) gene and frequency of blood types in stray cats of İzmir, Turkey

**DOI:** 10.1186/s12864-021-07588-0

**Published:** 2021-04-19

**Authors:** Hüseyin Can, Sedef Erkunt Alak, Ahmet Efe Köseoğlu, Umut Şahar, Berna Bostanbaş, Serdar Baydarlı, Mert Döşkaya, Cemal Ün

**Affiliations:** 1 Faculty of Science Department of Biology Molecular Biology Section, Ege University, Bornova, 35040 İzmir, Turkey; 2 Department of Veterinary Affairs, Municipality of Narlıdere, İzmir, Turkey; 3 Faculty of Medicine, Department of Parasitology, Ege University, Bornova, İzmir, Turkey

**Keywords:** *CMAH* gene, Blood typing, Stray cats, Turkey

## Abstract

**Background:**

*Cytidine monophospho-n-acetylneuraminic acid hydroxylase* (*CMAH*) gene associated with blood groups in cats encodes CMAH enzyme that converts Neu5Ac to Neu5Gc. Although variations in *CMAH* gene of pedigree cats have been revealed, the presence/lack of them in non-pedigree stray cats is unknown. Therefore, the present study aimed to investigate the variations in *CMAH* gene and the quantity of Neu5Ac and Neu5Gc on erythrocytes of non-pedigree stray cats (n:12) living in İzmir, Turkey. Also, the frequency of blood types was determined in 76 stray cats including 12 cats that were used for *CMAH* and Neu5A/Neu5Gc analysis.

**Results:**

In total, 14 SNPs were detected in 5’UTR as well as in exon 2, 4, 9, 10, 11 and 12 of *CMAH* gene. Among these SNPs, -495 C > T in 5’UTR was detected for the first time as heterozygous in type A and AB cats, and homozygous and heterozygous in type B cats. The remaining 13 that have been detected in previous studies were also found as homozygous or heterozygous. Both Neu5Gc and Neu5Ac were detected in type A and AB cats. In type B cats, only Neu5Ac was detected. Among two type AB cats, the level of Neu5Ac was found higher in cat carrying heterozygous form (T/C) of 1392T > C. The prevalence of type B cats (67.1 %) was higher than others.

**Conclusions:**

The presence of a new SNP as well as previous SNPs indicates that more variations can be found in stray cats with a more comprehensive study in the future. Also, the high prevalence of type B cats demonstrates the possible risk of neonatal isoerythrolysis among stray cats living in İzmir, Turkey.

## Background

The blood group system of cats was initially reported in the early 1900’s. In later years, three different blood types, called type A, B and AB, were demonstrated in cats. Genetic dominance among the three different blood types in cats has been reported as A > a^ab^> b. According to this, AA, Aa^ab^ and Ab genotypes can occur in Type A cats, a^ab^a^ab^ and a^ab^b genotypes in Type AB cats and bb genotype in Type B cats [1].

Determination of blood types in cats is important in veterinary clinical practice since blood type incompatibility causes transfusion reactions related to severe haemolytic anaemia, anaphylactic shock, and death [2]. In addition to transfusion reactions, neonatal isoerythrolysis can also occurred in cats when type A or type AB kittens are born from a type B queen [3]. Both transfusion reactions and neonatal isoerythrolysis are caused by high anti-A antibody levels found in B type cats [4, 5]. According to data from several studies associated with blood typing in cats, type A is the most prevalent blood group compared to type B and AB. For example, it has been reported in many surveys that more than 90 % of domestic cats are type A [6, 7]. The frequency of type B varies substantially from 0 to 59 % in the distinct geographic regions. In contrast to type A and B, the prevalence of type AB is generally less than 1 % worldwide [3].

The presence of different sialic acid residues on erythrocytes gives rise to different blood types in cats. Sialic acids that are expressed from echinoderms to mammals are mostly found as the terminal sugars of cell surface glycolipids and glycoproteins. There are more than 50 sialic acids derived from three main forms which are called N-acetylneuraminic acid (Neu5Ac), N-glycolylneuraminic acid (Neu5Gc) and 2-keto-3- deoxy-nonulosonic acid (KDN) [8]. Among them, the predominant sialic acids on most mammalian cells are Neu5Gc and Neu5Ac [9]. The Neu5Gc is expressed in a lot of mammals, except humans because of a deletion in the coding region of *CMAH* (cytidine monophospho-N-acetylneuraminic acid hydroxylase) gene [10]. Type A cats have mainly Neu5Gc and small amount of Neu5Ac, while type B cats have only Neu5Ac [11]. Type AB cats have both Neu5Gc and Neu5Ac at similar quantities [12]. It is stated that CMAH enzyme encoded by *CMAH* gene determines the type of sialic acid on erythrocytes by converting Neu5Ac to Neu5Gc in cats [13]. Accordingly, CMAH enzyme is active in type A cats while it is absent or nonfunctional in type B cats [1].

*CMAH* gene has been molecularly characterized among different cat populations and several single nucleotide polymorphisms (SNPs) (-539G > A, -468 A > G, -371 C > T, -217G > A, -108G > A, c.139 C > T, c.141 C > T, c.142G > A, c.179G > T, c.187 A > G, c.213 A > G, c.268T > A, c.327 A > C, c.364 C > T, c.374 C > T, c.376G > A, c.501G > A, c.593 A > C, c.636G > A, c.868 A > C, c.898 A > G, c.933 A > G, c.933delA, c.993 A > G, c.1158T > C, c.1218T > C, 1269G > A, c.1322delT, c.1342G > A, c.1392T > C, c.1398G > T, c.1452T > C, c.1458T > C, c.1603G > A, c.1662G > A) have been detected [1, 14–17]. In addition to these SNPs, there is an 18 bp insertion in 5’UTR region. Among these variations, some have also been associated with specific blood type/types. For example, 18 bp insertion has been reported to be specific for b allele found in type B cats as homozygous or in type A or AB cats as heterozygous [1].

There is no sufficient research about the presence/prevalence of these variations or their association with specific blood groups for stray cats of Turkey, except our previous study showing the presence of 18 bp insertion only in two of 791 stray cats [18]. Therefore, the present study aimed to investigate the presence of these variations in non-pedigree stray cats of İzmir, Turkey and their associations with blood group and the quantity of Neu5Ac and Neu5Gc on erythrocytes. To address these purposes, routine immunological blood typing was performed for 76 stray cats and among them, blood samples of 12 cats [A (n:5); B (n:5); AB (n:2)] were used for molecular characterization of *CMAH* gene.

## Results

### Prevalence of blood types

Among 76 stray cats, 52 of them were female (68.42 %), 24 were male (31.57 %). Conventional slide test showed that 23 of them were type A (30.26 %), 51 of them were type B (67.1 %) and the remaining two were type AB (2.63 %). There was not a statistical difference associated with prevalence of blood groups between female and male cats (*P > 0.05*).

### Polymorphisms in ***CMAH*** gene

*CMAH* gene analysis was performed using DNA samples belonging to 12 stray cats with known blood group (Table 1). During the *CMAH* gene analysis, 5’UTR region (including exon 1) as well as 14 exons (from exon 2 to exon 15 including 3’ UTR regions) were sequenced and analysed for SNPs. According to the results obtained, a total of 14 SNPs was detected in *CMAH* gene. The regions carrying SNPs were 5’UTR as well as exon 2, 4, 9, 10, 11 and 12 (Table 2). Among these SNPs, one of them caused by a cytosine-thymine substitution (-495 C > T) in 5’UTR was detected for the first time as heterozygous in type A and AB cats, and homozygous and heterozygous in type B cats. The remaining 13 SNPs that have been detected in previous study were also found as homozygous or heterozygous (Table 2). In addition, it was found that homozygous form of T1392C polymorphism was detected for the first time in type AB cat when compared to previous studies (Table 3). Interestingly, 18 bp insertion (AACGAGCAACCGAAGCTG) reported in 5’UTR region in type B cats was not detected in any type of cats.

**Table 1:** Blood group and some phenotypic results of analysed stray cats for characterization of *CMAH* gene

Sex	Color	Blood group
Female	Black	AB
Female	Black-White	AB
Female	Gray	A
Female	Calico	A
Female	Calico	A
Male	Tabby (White)	A
Female	Calico	A
Female	Black-White	B
Female	Tabby	B
Female	Tabby (White)	B
Male	Black	B
Male	Tabby (White)	B

**Table 2:** Polymorphisms detected in the *CMAH* gene in stray cats of İzmir

Blood type	5’ UTR				Exon 2			Exon 4	Exon 9	Exon 10		Exon 11		Exon 12
	^**a**^C-495T	**A-468G**^**b**^	**C-371T**^**b**^	**G-108A**^**b**^	**C139T**^**c**^	**G179T**^**d**^	**A187G**^**d**^	**A327C**^**e**^	**A993G**^**d**^	**T1158C**^**e**^	**T1218C**^**d**^	**G1269A**^**e**^	**T1392C**^**d**^	**T1458C**^**d**^
**AB**	CT	AG	CT	GA	CT	GT	AG	AC	AA	CC	TC	GG	TC	TC
**AB**	CT	AG	CT	GA	CT	GT	AG	AC	AA	CC	TC	GG	CC	TC
**A**	CT	GG	CT	GG	CT	GT	AG	CC	AA	CC	TC	GG	TC	CC
**A**	CT	GG	CT	GG	CT	GT	AA	CC	AA	CC	TC	GG	TC	CC
**A**	CT	AG	CT	GG	CT	GT	AG	CC	AA	CC	TC	GG	TC	CC
**A**	CT	AG	CT	GA	CT	GT	AA	CC	AA	CC	TC	GA	TC	TC
**A**	CT	AG	CT	GA	CT	GT	AG	CC	AA	CC	TC	GG	CC	TC
**B**	CT	AG	CT	GG	CT	GT	AA	CC	AG	CC	TC	GG	CC	TC
**B**	CT	AG	CT	GA	CT	GT	AA	CC	AG	CC	TC	GG	CC	TC
**B**	TT	GG	TT	GA	CT	GT	AA	CC	AG	CC	TC	GG	CC	CC
**B**	TT	GG	TT	GA	CT	GT	AA	CC	AA	CC	TC	GG	CC	CC
**B**	CT	AG	CT	GG	CT	GT	AA	CC	AG	CC	TC	GG	CC	TC

^a^shows the SNP that has been detected in this study for the first time

^b ^[1]

^c ^[17]

^d ^[15]

^e ^[14]

**Table 3:** Comparison of the SNPs detected in this study with previous studies

SNPs	The present study results			Previous studies results			References
	A	B	AB	A	B	AB	
C-495T	CT	CT, TT	CT	-	-	-	The present study
A-468G	AG, GG	AG, GG	AG	NP	NP	NP	[1]
C-371T	CT	CT, TT	CT	CC, CT	TT	CC, CT	[1]
				CC	CC, CT, TT	CC, CT	[15]
G-108A	GG, GA	GG, GA	GA	NP	NP	NP	[1]
C139T	CT	CT	CT	CC, CT	CC, CT	CC, CT	[17]
				CC	CC, CT	CC	[15]
				CC, CT	CC, CT, TT	CC, CT	[16]
G179T	GT	GT	GT	GG	GG, GT	GG	[15]
				GG, GT	GG, GT, TT	GG, GT	[16]
A187G	AA, AG	AA	AG	AA	AA, AG	AG	[15]
				AA, AG, GG	AA	AA, AG	[16]
A327C	CC	CC	AC	AA, AC	AA, AC, CC	CC	[15]
				AA, AC, CC	AA, CC	AA, AC, CC	[16]
A993G	AA	AA, AG	AA	AA, GG	AA, AG	AG, GG	[15]
				AA, AG, GG	AA, AG, GG	AA, AG	[16]
T1158C	CC	CC	CC	TT, TC	TC, CC	CC	[15]
				TT, TC, CC	TT, CC	CC	[16]
T1218C	TC	TC	TC	TT	TT, TC	TT	[15]
				TT, TC	TT, TC, CC	TT, TC	[16]
G1269A	GG, GA	GG	GG	GG	GG, GA, AA	GG, GA	[15]
				GG, GA	GG, GA, AA	GG, GA, AA	[16]
T1392C	TC, CC	CC	TC, CC	CC, TC	TT, TC, CC	TC	[15]
				TC, TT, CC	TT, TC, CC	TT, TC	[16]
T1458C	TC, CC	TC, CC	TC	TT, CC	TT, TC, CC	TT, TC	[15]
				TT, TC, CC	TT, CC	TT	[16]

*NP* Not present

An association of the detected polymorphisms with cat blood types were also analysed and homozygous form (T/T) of the − 495 C > T polymorphism detected in this study was found among only in type B cats. Among the polymorphisms previously determined in the literature, homozygous form of the − 371 C > T polymorphism was found among only in type B cats whereas heterozygous form (A/C) of the c.327 A > C polymorphism was detected only in type AB cats. In addition, only homozygous form of the c.1158T > C polymorphism were detected in all three blood groups.

### Type and amount of sialic acids in blood groups

During this study, Neu5Ac and Neu5Gc and their levels were also investigated in cats with known blood type using the LC-MS/MS system. According to the results obtained, both Neu5Gc and Neu5Ac were detected in type A and AB cats. In type B cats, only Neu5Ac was detected. *m/z* ratios and fragment ions detected for Neu5Ac and Neu5Gc were given in Table 4. The highest Neu5Ac level (4.53 µg/g) was among type B cats whereas the highest Neu5Gc level (4.39 µg/g) was detected among type A cats. When levels of Neu5Gc and Neu5Ac were compared within each blood group, the level of Neu5Ac was found higher in one of the two AB type cats. As these two cats were compared in terms of polymorphisms, AB type cat carrying homozygous form (C/C) of 1392T > C polymorphism in exon 11 had lower Neu5Ac than the other AB type cat carrying heterozygous form (T/C) of 1392T > C polymorphism. No association was found between the SNPs and the amounts of Neu5Gc and Neu5Ac detected in type A or B cats.

**Table 4:** Relative retention times and characteristic ions of Neu5Ac and Neu5Gc analysed by LC-MS/MS

			Collision-induced dissociation fragments	
Sialic acid	Retention Time	[M+H]^**+**^	[M+H-H_**2**_O]^**+**^	Fragments (m/z)
Neu5Gc	2,8	442	424	313-295-268-283-229
Neu5Ac	3,2	426	408	313-295-283-229

## Discussion

Determination of blood types in cats is important for clinical practices because of transfusion reactions and neonatal isoerythrolysis [2, 3]. Both transfusion reactions and neonatal isoerythrolysis are related to type B cats having high titer anti-A antibody [4, 5]. In our study, the prevalence of type B cats was found higher (67.1 %) when compared to type A cats. In Turkey, the prevalence results associated with type B vary according to cat breeds. For example, in a previous study, the prevalence of type B cats was found higher than type A cats among Turkish Van cats. Also, type B prevalence (46.4 %) was found nearly equal to type A (53.6 %) in Turkish Angora cats [2]. In another study, the prevalence of type B cats was found as 35.9 %, 32.6 %, 30.5 %, and 6.1 % in İstanbul, İzmit, Kırıkkale and Giresun, respectively, among non-pedigree cats [3]. These findings and our results indicate that there might be a possible risk for neonatal isoerythrolysis among pedigree cats (Turkish Van and Angora) and stray cats living in İzmir, İstanbul, İzmit and Kırıkkale provinces of Turkey.

*CMAH* gene that is associated with blood group system has been studied in cats for about 15 years. To date, several SNPs as well as an 18 bp insertion have been detected by sequencing *CMAH* gene. Among these variations, some of them have also been associated with specific blood types. For example, Bighignoli et al. (2007) reported the 16 SNPs as well as an 18 bp insertion in *CMAH* gene of cats and among these, -217G > A, -371 C > T, c.142G > A (originally G139A), c.268T > A (originally T265A), c.1603G > A (originally G1600A) and 18 bp insertion were found to be specific to blood group [1]. Accordingly, homozygous forms of these variations specific to type B cats or heterozygous forms could be found in b allele carrier cats such as heterozygous type A and AB cats. In our study, C-371T was detected in type A, B and AB cats as heterozygous (C/T). Moreover, homozygous form of the − 371 C > T polymorphism was found among only in type B cats. The remaining four SNPs (-217G > A, c.142G > A, c.268T > A, c.1603G > A) and 18 bp insertion were not detected in stray cats. These results demonstrate that there may be different variations in the *CMAH* gene between popular cat breeds and stray cats, and some of them are more common among popular cat breeds as well as being specific to blood groups. This assertion is actually supported by a study showing the presence of c.364 C > T polymorphism in only Ragdoll cat breeds with AB blood group [14].

In a different study, c.179G > T, c.187 A > G, c.1218T > C and c.1662G > A polymorphisms were detected in *CMAH* gene of type B cats as homozygous or heterozygous. c.187 A > G was also detected in type AB cats as heterozygous [15]. In our study, c.179G > T and c.1218T > C were found in type A, B and AB cats as heterozygous as well as c.187 A > G in only type A and AB as heterozygous. c.1662G > A was not found in stray cats. In the following study conducted by Kehl et al., (2018), a total of 29 SNPs including 13 new polymorphisms (c.141 C > T, c.213 A > G, c.374 C > T, c.376G > A, c.501G > A, c.593 A > C, c.636G > A, c.868 A > C, c.898 A > G, c.933delA, c.1322delT, c.1342G > A and c.1452 C > T) were detected in different cat breeds analysed. Among these SNPs, some of them were detected in homozygous or heterozygous forms in Turkish Angora cats [16]. The Turkish Angora cats that are one of the oldest cat breeds have originated from Ankara region of Turkey which is relatively close to İzmir, our study area. Therefore, previous results of Angora cats were compared with the analysed stray cats. Accordingly, homozygous or heterozygous forms of coding region polymorphisms (c.139 C > T, c.179G > T, c.187 A > G, c.327 A > C, A993G, T1158C, T1218C, G1269A, T1392C and T1458C) detected in our study were observed as coherent in both groups. Moreover, 18 bp insertion that has not been detected in our study was found only in one type B cat among eight Turkish Angora cats analysed [16]. These findings indicated that coding region polymorphisms detected in our study can be prevalent in Turkey whereas 18 bp insertion was rare. Our previous study showing the prevalence of 18 bp insertion as 0.25 % (2/791) also supports this result. [18].

Polymorphisms detected in *CMAH* gene have also been used as a genetic marker in the determination of blood groups. For example, Tasker et al. (2014) used two SNPs [G142A (original G139A) and C139T (original C136T)] to determine blood groups and reported that serological results were 96 % compatible with those of molecular methods [17]. Contrary to this, among these two SNPs, only one (C139T) was found in stray cats as heterozygous and did not show any correlation with blood groups. Therefore, the present results invalidate the genotype-blood group scheme for stray cats. -495 C > T that is detected for the first time in our study and − 371 C > T were found as homozygous (T/T) in same two type B cats. Also, heterozygous form (A/C) of the 327 A > C polymorphism was detected only in type AB cats. Although these results indicate a very limited relation with blood groups in stray cats, there is a need for additional studies analysing more stray cats with type B and AB. Moreover, no previously detected polymorphism which is known to be related to blood groups showed any exact relationship with blood groups in stray cats (Table 2).

In cats, types of sialic acids related to blood groups are known. Accordingly, type A cats have Neu5Gc while type B cats have Neu5Ac. Neu5Ac can also be found at low levels in type A cats. Type AB cats have both Neu5Gc and Neu5Ac at similar levels. In our study, types of sialic acid were found as coherent with literature in blood groups. However, among two AB cats analysed, Neu5Ac was found higher (nearly 4 folds) in one. As the two cats were compared in terms of polymorphisms, only single position (1392T > C) was different. Depending on this, it was thought that the homozygous form of the polymorphism (C/C) can change Neu5Ac level in type AB cats because SNPs found in promotor, intron or exon regions of any gene have potential to change the level of protein/enzyme expressions [19].

## Conclusions

The findings demonstrate the possible risk of neonatal isoerythrolysis among stray cats because of high prevalence of type B cats. In *CMAH* gene, identification of a new polymorphism (-495 C > T) and the presence of previously identified polymorphisms indicate that more polymorphisms can be found in stray cats with a more comprehensive study. Moreover, the results also indicate that polymorphisms that have been found as coherent with blood groups in previous studies are not acceptable for stray cats in this region.

## Methods

### Collection of blood samples

Blood samples were collected from healthy stray cats (n:76) which were brought to Veterinary Clinics in Narlıdere province of İzmir, Turkey for sterilization purposes. 1–2 ml of blood sample was obtained from tissue material that was removed from the anesthetized cats during sterilization, inserted into 5 ml tubes with EDTA and kept at + 4 °C until used.

### Blood typing

Blood typing was performed for 76 blood samples by conventional slide test as described [20]. Briefly, a serum sample belonging to type B cat as anti-A reagent and Lectin *Triticum vulgaris* (1 mg/ml) as anti-B reagent were used. 50 µl of anti-A and anti-B reagents were individually added to a slide and both of them were gently mixed with 25 µl of blood sample by a pipette. After 5 min, blood types were detected by observing the presence of agglutination. Among 76 blood samples, 12 of them [A (n:5); B (n:5); AB (n:2)] were also studied by a commercial immunochromatographic strip method [Alvedia rapid-test (LabTest A + B) to confirm blood type results. These confirmed samples were used for analysis of *CMAH* gene.

### PCR

DNA isolation from blood samples was conducted by PureLink Genomic DNA Mini Kit (Thermo Fisher Scientific) according to the manufacturer’s instructions. During PCR, 16 different regions of *CMAH* gene including a 5’ UTR and 14 exons as well as 3’UTR were amplified using primer pairs [14, 15]. PCR mixture contained 2 µl template DNA, 12.5 µl Dream Taq master mix (Thermo scientific), 1 µl from each of primers (10 pmol) and 8.5 µl distilled water. The PCR amplifications were carried out under the following conditions: 2 min initial denaturation step at 95 °C, followed by 35 cycles of 1 min at 95 °C, 45 s at 58 °C, and 45 s at 72 °C, and a final extension of 10 min at 72 °C [1]. PCR products were run through 1 % agarose gel electrophoresis and visualized.

### Sequencing

PCR products belonging to *CMAH* gene were sequenced by ABI3730XL and generated sequences were aligned by MEGA7.0 software to compare exons belonging to reference *Felis catus CMAH* gene with accession number NM_001244985.1 and to compare 5’ UTR regions belonging to reference *Felis catus CMAH* genes with accession numbers EF127683 and EF127686.

### Preparation of the samples and LC-MS/MS analysis

The determination of Neu5Ac and Neu5Gc in erythrocyte samples isolated from whole blood was carried out using modification of the methods reported by Hara et al. (1986) and Yeşilyurt et al. (2015) [21, 22]. Briefly, 30 mg erythrocyte sample was incubated for 90 min at 80 ^o^C with 0.1 M trifluoroacetic acid (TFA) for acid hydrolysis in order to release the sialic acids. After that, released sialic acids were derivatized with DMB (1,2-Diamino-4,5-methylenedioxybenzene dihydrochloride) solution containing 1.55 mg DMB, 3.68 mg sodium hydrosulfite and 50 µl 2-mercaptoethanol prepared in 950 µl 0.1 M TFA. Derivatized sialic acids were pipetted into the HPLC vial insert. The injection volume was settled to 0.3 µL in the method. Commercial Neu5Ac (Sigma) and Neu5Gc (Sigma) were used as standard.

HPLC analysis was performed using an Agilent 1200 Capillary HPLC system with an ODS capillary column (Agilent ZORBAX SB-C18 150 0.5 mm, 5 mm, USA). Elution was performed by isocratic mode at 20 µL/min using a mixture of (methanol/acetonitrile, 3:2) and water at 1:4 ratios. The column temperature was kept at 30℃ during the analysis. All mass spectrometric measurements were performed on an HCT Ultra ion trap mass spectrometer (Bruker Daltonics, Bremen, Germany) equipped with an electrospray ionization (ESI) source in positive mode. Ion optics voltages, nebulizer gas, and dry gas flow rates, and the dry gas temperature were controlled by EsquireControl software 6.1. All mass spectra were acquired in the mass range 200–600 m/z, with a scan speed of 26,000 m/z per second. Data analysis was carried out using Data Analysis software (v.3.4, Bruker Daltonics).

## Data Availability

All sequences obtained from *CMAH gene* of stray cats were deposited into GenBank (National Center for Biotechnology Information Search database). Provided GenBank accession numbers are as follows. MW750673, MW750674, MW750675, MW750676, MW750677, MW750678, MW750679, MW750680, MW750681, MW750682, MW750683, MW750684, MW750685, MW750686, MW750687, MW750688, MW750689, MW750690, MW750691.

## Abbreviations

CMAH: Cytidine monophospho-n-acetylneuraminic acid hydroxylase
Neu5Ac: N-Acetylneuraminic acid
Neu5Gc: N-Glycolylneuraminic acid
5’UTR: 5′ untranslated region
KDN: 2-keto-3- deoxy-nonulosonic acid
SNPs: Single nucleotide polymorphisms
A: Adenine
C: Cytosine
G: Guanine
T: Thymine
TFA: Trifluoroacetic acid
DMB: 1,2-Diamino-4,5-methylenedioxybenzene dihydrochloride
